# Perception of simulation-based training as a replacement for clinical practice training among pre-licensure nursing students: a qualitative study

**DOI:** 10.1186/s12912-025-04096-4

**Published:** 2025-12-01

**Authors:** Ka Ming Chow, Anna Hau Yi Ngan, Conny Sau Man Chan

**Affiliations:** The Nethersole School of Nursing, The Chinese University of Hong Kong, Shatin, New Territories, Hong Kong SAR, China

**Keywords:** Clinical practice, Emergency nursing, Higher education, High-fidelity, Nursing education, Simulation

## Abstract

**Background:**

Despite the increasing adoption of simulation during clinical training disruptions, little is known about how students perceive its ability to replace clinical placements. This study aims to explore the perceptions, attitudes and experiences of final-year pre-licensure nursing students regarding simulation-based training as a substitution for clinical practice.

**Methods:**

This study adopted an exploratory qualitative design with face-to-face focus group interviews. Two accident and emergency case scenarios involving tachycardia and drug overdose were developed and simulated in the simulation wards of the participating nursing institution to substitute for clinical practice during the COVID-19 pandemic. We recruited a total of 40 final-year pre-licensure nursing students (80% female) enrolled in both bachelor’s (*n* = 20) and master’s programs (*n* = 20) in a government-funded public university in Hong Kong to participate in focus group interviews via convenience sampling until data saturation was reached. All interviews were audio-recorded and moderated by nurse instructors using a pre-determined interview guide. Qualitative data were subjected to thematic analysis.

**Results:**

A total of eight focus group interviews were conducted. Three themes emerged from the qualitative data: (i) effective preparation for clinical practice, (ii) the limitations of simulation-based training in replacing clinical practice and (iii) refining simulation-based training. All students recognised that the simulation-based training helped to bridge the gap between theory and practice and to improve their case management, communication, clinical decision-making and nursing skills. However, limitations concerning the differences in procedures and facilities not reflective of the most current practices and challenges in replicating certain aspects of clinical placement (such as clinical atmosphere, patient variety and complexity) hindered the ability of simulation-based training to fully replace clinical practice.

**Conclusion:**

The study findings demonstrate the potential use of simulation-based training to complement clinical practice in pre-licensure nursing education in Hong Kong. To further refine the training programme, it is recommended to enhance the briefing, develop more complex and diverse case scenarios to better replicate clinical situations and upgrade the laboratory facilities to include more realistic equipment. Further research should explore the potential use of this training modality in other speciality areas and broader variety of institutions using a longitudinal design.

**Clinical trial number:**

Not applicable.

**Supplementary Information:**

The online version contains supplementary material available at 10.1186/s12912-025-04096-4.

## Background

In higher education, students acquire subject-specific knowledge and develop complex skills such as critical thinking and problem solving, equipping them to make informed professional decisions in their future careers [1]. However, higher education often lacks opportunities for practicing complex skills in real-life situations. To overcome these challenges, approximations of practice, such as simulations, are designed to allow students to engage with real-life problems and effectively develop complex skills in a controlled and safe environment [1]. Since its introduction in the late 1980s, simulation-based training (SBT) has become an innovative approach to medical education that allows students to practice skills and mimic real-world clinical scenarios in a laboratory, without harming patients. In nursing education, simulation is ‘a dynamic process involving the creation of a hypothetical opportunity that incorporates an authentic representation of reality, facilitates active student engagement and integrates the complexities of practical and theoretical learning with opportunity for repetition, feedback, evaluation and reflection’ [2]. Empirical studies have shown that the use of high-fidelity simulation can benefit students’ learning. Specifically, the use of high-fidelity simulation can improve the acquisition of knowledge and psychomotor skills [3, 4]. This teaching modality enhances the problem-solving skills, self-efficacy and clinical competency of nursing students [3, 5–7]. A literature review indicated that multiple-patient simulation improved nursing students’ perceived readiness for practice and teamwork [8].

The design, implementation, and evaluation of SBT can be guided by the National League for Nursing (NLN) Jeffries Simulation Theory, which was developed through robust research and literature review, as well as insights from nursing simulation experts [9]. This theory features six core elements - context, background, design, simulation experience, facilitator and education strategies, participant, and outcomes [9]. Outcomes are grouped into three main areas including participant, patient and system outcomes. In this study, we focused on participant outcomes [9].

The increasing complexity of the healthcare environment, the growing number of nursing students and a shortage of clinical supervisors have increased the pressure associated with clinical placement opportunities. The use of SBT to substitute a portion of traditional clinical placement hours has been increasingly researched and adopted by nursing programmes [10, 11]. A national trial study in the United States (US) found no statistically significant differences in clinical competency, comprehensive nursing knowledge, readiness for practice and self-confidence between nursing students who received traditional clinical placement and those for whom either 25% or 50% of their clinical hours were replaced by SBT [12]. Regulations regarding the percentage of clinical hours that can be replaced by SBT differ across jurisdictions. For example, Singapore allows a maximum of 25% of clinical hours to be replaced by SBT [13], whereas some US states allow up to 50% of clinical hours to be replaced [14].

The role of SBT became more crucial during clinical training disruptions. For example, during the COVID-19 pandemic, strict infection control measures prevented students from completing clinical practicum hours in high-risk clinical areas, including accident and emergency (A&E) departments, infectious disease wards, paediatrics departments, intensive care units and mental health departments. However, clinical learning is an integral component of pre-licensure nursing education. Such limitations would affect nursing students’ ability to graduate and the licensure of registered nurses. SBT thus has become a reasonable alternative to clinical placement that ensures the safety of both learners and patients [15]. To this end, nursing schools faced unprecedented challenges in rapidly designing and implementing high-quality, face-to-face or virtual simulations to substitute for clinical placements during the COVID-19 pandemic [16, 17]. Although SBT was rapidly adopted, little is known about how nursing students perceived its use as a substitute for clinical practice, particularly in high-risk specialties.

In Hong Kong, clinical placement in certain specialty areas has resumed since the end of 2020 with the alleviation of the pandemic situation. However, only limited numbers of places are offered in most specialty areas. To provide clinical training for nursing students and allow them to fulfil the requirements for graduation and professional registration, regulatory allowances were introduced for the first time in Hong Kong, to replace some of the clinical training hours with alternative modes of delivery, including SBT. Prior to the pandemic, there were no guidelines regarding the use of SBT to substitute for the clinical training hours required for nursing licensure in Hong Kong. The current arrangement represents a significant milestone in local nursing education. However, nursing students’ perceptions of this mode of training as a replacement for clinical practice remain unclear. This study explored the perceptions, attitudes and experiences of final-year pre-licensure nursing students regarding the replacement of clinical practice by SBT. Students’ perceptions are essential for future curriculum planning. The findings provide important insights for nursing educators on how to effectively design and implement SBT to enhance students’ learning outcomes.

## Methods

### Study design

This study used an exploratory qualitative design and face-to-face focus group interviews to facilitate discussion and shared reflection among students. This study report adheres to the Consolidated Criteria For Reporting Qualitative Research [18].

### Setting and participants

The study setting is a government-funded public university in Hong Kong that offers pre-registration nursing programs at both the bachelor’s and master’s levels. All final-year students in the Bachelor of Nursing (BN) programme and Master of Nursing Science (Pre-registration) Programme (MNSP) in the Nethersole School of Nursing, the Chinese University of Hong Kong were eligible to participate. The sample pool for this study comprised 318 Chinese students. We conveniently recruited an equal number of students (*n* = 20) from each programme to participate in the focus group interviews until data saturation was achieved for each programme. Convenience sampling was chosen due to easy accessibility and time constraints.

### High-fidelity simulation training

Two A&E case scenarios involving tachycardia and drug overdose were developed and simulated in the simulation wards of the participating nursing institution. Each scenario was delivered in a half-day session with an approximate duration of three hours, which was divided into briefing, participation and debriefing. Each session was moderated by one nurse instructor who had received a four-day comprehensive simulation educator course in simulation education, scenario design and debriefing skill for facilitating SBT. The students were divided into groups of eight and further into two sub-groups. The two groups rotated between participation and observation. The students were required to pre-read the simulation training materials in advance. The instructor assigned roles to each student during the briefing sessions. The students were required to actively participate in the simulation activities. They were expected to demonstrate critical decision-making skills when providing client-centred care based on the simulation scenarios. In the debriefing phase, the instructor explained the scenario and reflected on the experience with the students. The instructor also assessed the students’ performance. All nursing students participated in SBT as a replacement for 16 h of clinical practice in the A&E specialty during the study period.

### Data collection

All participants were invited to participate in Cantonese-language focus group interviews after completing SBT. Focus interviews are commonly used in nursing education research considering the advantage of increased participant interaction, which enhances data reliability as participants can support or question each other’s comments [19]. Interviews were conducted until data saturation was reached i.e. no new codes or themes emerged in two consecutive interviews. Three instructors (one male and two female) with substantial experience in conducting qualitative interviews with nursing students moderated the interviews in the simulation laboratory, using an interview guide as a reference (Supplementary Material 1). The interview questions explore perceptions, attitudes and experiences regarding SBT, including its potential to replace clinical hours, its influence on clinical competency development, enjoyable and challenging aspects of the experience. All the interviews were audio-recorded.

### Data analysis

The demographic data of the participants were summarised using descriptive statistics. The focus group interviews were transcribed verbatim and verified by the research team against the audio recordings. Qualitative data were subjected to thematic analysis using NVivo 2020 (QSR International, US) [20]. First, two researchers (A and B) independently read all the transcripts to identify the initial codes. Next, they condensed similar codes into subthemes and themes through iterative discussions. The team then continuously compared and discussed the subthemes and themes and refined them until a consensus was reached [20]. Finally, the codes, subthemes, themes and sample quotations were translated by researcher A, who is fluent in both English and Cantonese. Researcher B reviewed the translations to ensure semantic equivalence.

### Trustworthiness

Lincoln and Guba [21]’s criteria of credibility, dependability and confirmability were adopted to ensure a rigorous study. To enhance credibility, we provided representative quotes and outlined the interviewer’s role and experience, and two researchers independently conducted the analysis and reached consensus on the final themes, subthemes and codes [22]. To enhance dependability, all focus group interviews were guided by the same interview guide (Supplementary Material 1) [22]. Finally, to maximise confirmability, we presented a comprehensive collection of interview quotes, organised according to their respective codes, subthemes and overarching themes, in Supplementary Material 2 [23].

### Ethical considerations

Ethical approval for this study was obtained from The Chinese University of Hong Kong Survey and Behavioural Research Ethics Committee (Reference No. SBRE-22‐0169). All participants received a study information sheet and provided written informed consent before participating in the interviews. Participation in this study was voluntary, and the students had the right to withdraw from the study at any time without any adverse effect on their academic studies. All participants were assured their confidentiality in the study, and their names were not recorded. Each participant was assigned a unique numerical code to protect their identifiable information. All data, including audio recordings and transcripts, were stored securely in a password- protected computer accessible only to the research team. Findings were reported using numerical codes to ensure no individuals could be identified.

## Results

We recruited 20 bachelor’s students (mean age: 22.1 ± 0.6 years) and 20 master’s students (mean age: 26.1 ± 2.6 years) to participate in qualitative focus group interviews. Each interview lasted 45–60 min. The majority of the students were female (80%). All but one student had 4–30 months of experience in working as a temporary undergraduate nursing student in public hospitals, and 40% had experience working in medical wards. Eight focus group interviews were conducted, each consisting of five students enrolled in either the bachelor’s or master’s programme. Table 1 summarises the characteristics of the participants.

In the thematic analysis, three themes were generated from the qualitative data: (1) effective preparation for clinical practice (2) the limitations of SBT in replacing clinical practice and (3) refining SBT. Descriptions of the themes and illustrative quotes are provided below. Supplementary Material 2 outlines the themes, subthemes, codes and additional sample quotes.

### Theme 1: effective preparation for clinical practice

All students perceived that SBT helped to prepare them for clinical placement or post-registration practice because it could help to bridge the gap between theoretical knowledge and practice and improve their skills.

**Table 1:** Characteristics of participants

Characteristics	Bachelor of Nursing (*n* = 20) Mean ± SD / *n*(%)	Master of Nursing Science (Pre-registration) (*n* = 20) Mean ± SD / *n*(%)	All (*N* = 40) Mean ± SD / *n*(%)
Age (years)	22.1 ± 0.6	26.1 ± 2.6	24.1 ± 2.7
Gender			
Female	16 (80.0%)	16 (80.0%)	16 (80.0%)
Male	4 (20.0%)	4 (20.0%)	4 (20.0%)
Having experience in working as a TUNS			
No	1 (5.0%)	0	1 (2.5%)
Yes	19 (95.0%)	20 (100.0%)	39 (97.5%)
Duration of working as a TUNS (months)	15.2 ± 7.1	9.0 ± 1.3	12.1 ± 5.8
TUNS specialty			
Medical	10 (52.6%)	7 (35.0%)	17 (43.6%)
Accident & Emergency	0 (0%)	2 (10.0%)	2 (5.1%)
Others	9 (47.4%)	11 (55.0%)	20 (51.3%)

SD: Standard Deviation; TUNS: Temporary undergraduate nursing student in the public hospitals

#### Subtheme 1.1: bridging the gap between theoretical knowledge and practice

During SBT, the students learnt how to apply their theoretical knowledge in a clinical setting. They highlighted a few characteristics of SBT that facilitated this connection, including realistic case scenarios, active participation, opportunities for trial and error and reflective learning.

*I think the good thing about SBT…aside from what my classmates have mentioned about role play and communication…it’s a really great opportunity to gain a certain degree of practical experience because sometimes we often spend a lot of class time just on lectures. After you go through the materials and memorise them*,* that’s it. Even in the lab*,* you may just get a taste of things*,* like only trying out how to palpate or auscultate. (Student 1*,* Bachelor’s group 4)*

*I think the best aspect of the simulation lab*,* and what I found most enjoyable*,* is that we can apply the skills we’ve learnt in lectures. For example*,* we can practice nursing communication skills from Year 1. Communication is very important*,* whether we’re working as a team or talking to patients. SBT lets us practice these skills*,* and it’s quite…it’s an impressive experience. (Student 4*,* Master’s group 2)*

##### Realistic case scenarios (*n* = 18)

The majority of the students considered the SBT experience to be realistic and to highlight the intense atmosphere. In particular, some were impressed by how they recalled and applied the procedures practised in SBT when facing similar scenarios during their clinical practicum. Regardless of whether their clinical practicum was before or after SBT, the students expressed confidence in their understanding of the nurse’s role when they encountered similar scenarios in real clinical settings.

*I totally agree with Student 5 about how we gain hands-on experience and practise making decisions. For example*,* when we were working on trauma cases and had to do tapping or decompression*,* I rushed to grab the equipment as soon as I heard that we might need it. During preparation*,* I thought about how the whole process should be done. That experience helped me a lot. Also*,* the equipment at our school seems quite complete and similar to what you’d find in a real setting*,* a real environment. You can find whatever you need right away*,* which is similar to real-life situations. I think this is awesome because it helps me learn to think on my feet and it really prepares me for my clinical placement. (Student 1*,* Master’s group 2)*

Regarding the case scenarios, many students appreciated the opportunity to engage with more complicated and critical scenarios, which they would not be able to observe in a clinical practicum. This exposure enhanced their confidence in managing similar cases in real clinical settings.

*SBT provided a valuable supplementary experience because I didn’t get to see the tube procedure in clinical placement. I remember there was a trauma case*,* although I can’t recall if it was a trauma case or a drug overdose case. Anyway*,* there was a situation that required intubation. Even though I didn’t see it in A&E*,* at least I got to experience it in SBT. (Student 3*,* Bachelor’ s group 2)*

##### Active participation (*n* = 28)

During clinical practice, the students often held runner or observer roles with limited active involvement. They appreciated that the ability to actively participate in SBT, which allowed them to apply what they had learnt from their textbooks. Some students commented that this mode of learning was more effective than traditional methods such as lecture-based teaching or recitation.

*SBT is more student-led. If we have questions*,* we ask the tutors and [they] give feedback to us. It’s not like in lectures*,* where the lecturer spoon-feeds the knowledge. (Student 5*,* Bachelor’s group 1)*

The students appreciated the opportunity to assume more advanced roles, such as those of case or in-charge nurses, during SBT. These roles allowed them to actively engage in case management and enhanced their understanding of their clinical responsibilities. The students felt that this experience better prepared them for real-world clinical practice.

*I think this is an enjoyable experience because we can try out different roles even if we don’t have any experience. For example*,* I played the role of an IC (in-charge) nurse. Without a certain level of experience*,* we wouldn’t be able to take on that role in a real clinical setting… When your role changes*,* you cannot just think like a runner anymore… During SBT*,* we can do things that we normally would not get to do [in clinical placement]. (Student 4*,* Master’s group 4)*

##### Opportunities for trial and error (*n* = 30)

Knowing that they would not harm real patients, the students felt less pressure and were better able to try and learn from the mistakes. As trial and error is not acceptable in a clinical practicum or post-registration practice, they appreciated the opportunity to learn using this approach in SBT. Several students noted that learning from failure allowed them to master skills and avoid mistakes in real clinical settings.

*I think giving students more autonomy in SBT is a good thing… With such autonomy*,* even if we don’t know how to do something or make mistakes*,* I think the trial-and-error experience helps deepen our memory and teaches us how to prioritise actions in critical cases next time. (Student 3*,* Bachelor’s group 3)*

##### Reflective learning (*n* = 30)

Most students acknowledged the importance of debriefing sessions led by their tutor, which facilitated self-reflection on their performance and helped them to identify areas for improvement.

*I think that the debriefing session is quite important because*,* based on my experience*,* the tutor initially lets us try things out on our own. The two groups rotate. The first group usually is a bit clumsy. After observing the first group*,* the second group may develop some skills and figure out how to do things better. So*,* I think that the debriefing session is even more important because it helps us identify areas for improvement. The tutors let us try first. (Student 2*,* Bachelor’s group 2)*

#### Subtheme 1.2: improving skills

The design of SBT enables students to engage in comprehensive case management, collaborate as a team and make informed clinical decisions. The students perceived that SBT enhanced their case management, communication skills, clinical decision-making skills and nursing skills.

##### Case management (*n* = 39)

SBT is structured to guide students through the case management process, which is not possible in a busy clinical environment. Students found the step-by-step approach in SBT effective in terms of improving their understanding of comprehensive case management.

*I think one advantage of SBT over clinical placement is that it provides a more comprehensive view of the case. For example*,* we can role play as nurses and practice handing over a patient to another specialty. This is not something we can do in a real A&E setting. By simulating the entire process*,* we gain a better understanding of what it means to be a nurse and how to handle a case throughout the process. During clinical placement in A&E*,* our exposure to specific knowledge is often limited to what we observe. (Student 1*,* Bachelor’s group 4)*

##### Communication skills (*n* = 13)

During SBT, the students engaged in collaborative tasks that emphasised teamwork. This enhanced their understanding of the importance of clear and concise communication when interacting with doctors and other nurses in A&E.

*These skills can be applied in practice*,* and communication is very important*,* both in teamwork or when talking to patients. These aspects can be learnt and practiced in SBT*,* making it an impressive experience. (Student 4*,* Master’s group 2)*

##### Clinical decision-making skills (*n* = 34)

The simulation design allows ample time for preparation, discussion and reflection on each scenario. According to the students, this feature allowed them to critically analyse and make informed decisions in a safe environment, which they could later apply when dealing with similar cases during a clinical practicum.

*When we make clinical decisions in SBT*,* it involves careful thinking. We need to understand the case*,* the situation*,* which treatments or medications can help and any medications that might make it worse… We need to be clear about these things before making decisions. After learning about medications*,* we will be able to spot any mistakes*,* such as if doctors give wrong orders in real A&E settings. We won’t just blindly follow orders. We can use critical thinking based on solid knowledge. (Student 4*,* Bachelor’s group 3)*

##### Nursing skills (*n* = 10)

Some students perceived that SBT allowed them to focus on practising proper nursing procedures, such as medication administration and injections. These hands-on practices improved their nursing skills.

*I think SBT can enhance our nursing skills*,* such as administering injections. In a real hospital setting*,* the staff are often quite busy and may not have the time to supervise us whilst we distribute medications. As a result*,* there are fewer opportunities to give injections and distribute medications during our placement. (Student 5*,* Bachelor’s group 1)*

#### Subtheme 1.3: SBT as an alternative to clinical practice in special circumstances (*n* = 18)

Nearly half of the students agreed that SBT could be an alternative to practice nursing roles if a clinical practicum is not possible. For example, during the COVID-19 pandemic, the clinical practicum requirement for nursing students in hospitals under the Hospital Authority was suspended in high-risk areas such as A&E departments.

*Yes*,* I am very grateful for SBT*,* which allows us…even if we can’t experience the resuscitation room in person*,* we can still get a sense of the situation there. I think this helps fill the gap in our training. (Student 1*,* Master’s group 4)*

### Theme 2: limitations of SBT in replacing clinical practice

Most students argued that SBT cannot fully replace clinical practice due to several limitations, including differences in procedures and facilities and the challenges of replicating certain aspects of clinical training.

#### Subtheme 2.1: differences in procedures and facilities

##### Procedural differences (*n* = 9)

Although the students appreciated the opportunity to practice procedures properly during SBT, they perceived that these procedures might not be practical in a busy clinical setting such A&E. During their clinical practicum, some students observed that the clinical nurses used more effective or updated methods to achieve the same objectives. As a result, they believed that clinical placement provided valuable opportunities to learn the most current practices from experienced nurses.

*Whilst our teachers in SBT are very experienced*,* the unique environment of A&E…being surrounded by a team of specialised staff allows us to learn even more. Some techniques we learnt from books may be theoretical*,* but in practice [the nurses] might take shortcuts or use more effective methods. It’s important to learn those methods during placement. (Student 5*,* Bachelor’s group 3)*

##### Differences in facilities and equipment (*n* = 25)

Some students criticised the laboratory facilities and equipment, which they considered to be outdated and not reflective of the most current practices used in real clinical settings. They valued the opportunity to learn about the most current facilities and equipment during their clinical placement.

*Regarding the equipment*,* for example*,* the prime solutions are mostly just regular drips. We rarely get to use the machines. I’ve heard rumours that the equipment is expensive or that there isn’t enough*,* so we seldom get asked to administer*,* for example*,* Q150*,* 500 mL of normal saline. This means that we miss out on chances to practice using those machines. (Student 4*,* Master’s group 1)*

#### Subtheme 2.2: challenges of replicating certain aspects of clinical training

Whilst the simulations attempted to deliver realistic learning experiences, the students identified several aspects of real clinical settings that were challenging to replicate. These include the clinical atmosphere, interactions with real patients and families, patient variety and complexity, patient flow and unpredictability.

##### Clinical atmosphere (*n* = 34)

Most students commented that their SBT experience was very smooth because the clinical setting, patients and staff were well controlled. They did not feel pressure or urgency to act. In contrast, they commented that the atmosphere in a real A&E setting was much more intense and chaotic, which might negatively affect their performance.

*I strongly agree with what Student 2 said. The atmosphere in SBT is very different from that in an actual clinical setting. The real clinical setting is much busier*,* and when we’re in SBT practising with manikins*,* it feels less urgent. This can lead to a slower mindset or reduced focus. However*,* in real situations*,* we should maintain a more urgent and cautious attitude. Overall*,* the two environments are quite different. (Student 5*,* Master’s group 2)*

##### Interaction with real patients and families (*n* = 36)

As the patients and family members in the simulation sessions were not real, the students were unable to practice communication skills in challenging situations, particularly when interacting with challenging patients or family members. Furthermore, some students commented that the simulation manikin could not respond to nursing care in the same way as a real person. For example, one student shared an experience in which a patient complained of pain whilst receiving an injection.

*In A&E*,* one particularly memorable experience was when I was giving an intramuscular injection*,* and a patient said*,* “That hurts so much*,* I’m bleeding all over.” In SBT*,* you wouldn’t have a real patient telling you that your technique is poor or complaining like that. Although it was just a minor comment*,* it made a lasting impression on me. In SBT*,* I don’t think we would come across such a situation. We might hear something similar from a tutor*,* but we’d be likely to forget it later. But I’ll always remember that moment because it was a real patient speaking to me. (Student 3*,* Bachelor’s group 3)*

##### Patient flow (*n* = 33)

SBT typically focuses on specific skills or situations and addresses only part of the overall case management process. Some students indicated that the entire patient flow in A&E, including admission, triage, doctor consultation and multidisciplinary involvement, could only be observed or practiced during a real clinical placement.

*In SBT*,* it’s hard to replicate the experience you gain during placement*,* where you can follow a mentor for a day and see how they manage each case. There are many logistical tasks*,* such as knowing which patients need a chest X-ray or blood tests. I think we can handle those logistical aspects in clinical placement or clinical settings*,* but in SBT it’s too complicated*,* and there’s no system for us to click and see that information. Also*,* there’s no multi-disciplinary team involvement in a simulation. (Student 4*,* Master’s group 4)*

##### Patient variety and complexity (*n* = 27)

The students perceived that the case scenarios typically focused on only one critical condition. In contrast, real clinical settings often involve patients with less critical conditions, such as minor surgery or multiple comorbidities.

*In SBT*,* we practice on very critical cases*,* but in my clinical placement*,* I often find myself assisting nurses with minor surgeries. These experiences are usually not available in SBT. Additionally*,* many techniques and technical skills*,* such as the proper injection techniques and the order of actions that we encountered in clinical placement*,* also have a significant impact on our learning. (Student 1*,* Bachelor’s group 3)*

##### Unpredictability (*n* = 16)

Some students highlighted the inability of SBT to fully replicate the unpredictable nature of real-world clinical practice. Unlike the controlled setting of SBT, real clinical environments present unforeseen challenges and situations that require quick responses and high adaptability.

*SBT may only provide knowledge input*,* whilst real-life environments are better for developing your on-the-spot problem-solving skills*,* as many situations are ad hoc. (Student 1*,* Bachelor’s group 4)*

### Theme 3: refining SBT

Some students proposed several areas for improvements regarding SBT design and upgrading facilities and equipment to address the current limitations and create a more realistic learning experience. These improvements were deemed to be necessary if simulation is intended to replace some clinical training hours.

#### Subtheme 3.1: design of SBT

##### Enhance the briefing (*n* = 15)

Many students initially found SBT confusing: they felt lost even after completing the required pre-readings. This confusion affected their performance during the simulation sessions. To avoid wasting time and effort, some students suggested extending the initial pre-simulation briefing to allow students to discuss the scenarios with their peers and tutors.

*I also agree with what Student 3 said about our first attempts: whether we were doing IC or different roles*,* everyone was lost regarding their responsibilities. This is partly because it had been a long time since we had completed automated external defibrillator (AED) training*,* and also because when we get to a scene*,* we may not fully understand the responsibility of every role…I might make mistakes*,* and learning from them is valuable. But I believe that if we had a bit more briefing at the beginning*,* like a video demonstration*,* it would help us to get to know the flow. Alternatively*,* having a briefing on the responsibilities of roles such as the IC*,* along with a general flow of the triage process*,* would give us some guidance to work from. (Student 1*,* Master’s group 4)*

##### Involve clinical nurses (*n* = 7)

Several students raised concerns about the expertise of the facilitators, who were not familiar with the A&E setting. They proposed inviting practising nurses to act as the facilitators or to lead sharing sessions, where they could offer valuable insights and guidance, thus enriching the learning experience with their expertise.

*I was wondering whether there could be a bit more sharing*,* perhaps by inviting nurses who have worked in certain departments or specialties to speak. I think it’s really difficult to replicate their experiences in a simulation. (Student 2*,* Bachelor’s group 2)*

##### Increase case variety and complexity (*n* = 25)

To enrich the realism of SBT, a majority of the students recommended expanding the range of case scenarios to increase the case variety and complexities. Subsequently, some students suggested increasing the number of sessions to accommodate this enhancement.

*Regarding the multi-tasking I mentioned earlier*,* it would be great if we could have several manikins at the same time*,* each presenting a different case. For instance*,* SBT could allow us to handle multiple cases within one hour – not just trauma cases*,* but also situations like eye washing or fever. This would allow students to manage different cases at the same time and see how they handle multitasking. I think this could be a more effective approach. (Student 2*,* Master’s group 2)*

#### Subtheme 3.2: upgrading facilities and equipment (*n* = 6)

To address limitations related to the facilities and equipment, some students suggested upgrading the facilities and equipment in the simulation laboratory to more closely resemble a real clinical environment, and providing students with hands-on experience using the current facilities and equipment.

*Like earlier*,* when we were trying to find a surgical pack to stop the bleeding*,* it seemed that the only available equipment was combined gauze*,* which is a bit different from what we usually see during a clinical placement. If we could have more supplies*,* it would greatly enhance this experience. (Student 2*,* Master’s group 1)*

## Discussion

This is one of the first qualitative studies in Hong Kong to explore the perceptions of final-year pre-licensure nursing students regarding the use of SBT as a substitute for clinical practice. Overall, the students acknowledged that SBT could effectively prepare them for clinical practice. However, they noted that the limitations of the simulation made it difficult to fully replace clinical training in a real-world clinical setting. Some suggestions were made to improve SBT. It is important to note that the findings of this study may have been influenced by the unique context of the COVID-19 pandemic. The stringent infection control measures have driven increased adoption of online learning and simulation, which may have influenced students’ experiences and attitudes in ways that are not representative of a non-pandemic context. Nevertheless, in view of the increasing the pressure associated with clinical placement opportunities and the increasing adoption of SBT to substitute for traditional clinical placement hours, our study findings provide important insights on how to effectively design and implement SBT to enhance students’ learning outcomes.

In this study, all the students perceived that SBT better prepared them for clinical practice because it served as a bridge between theoretical knowledge and practice and thus improved their skills. This finding is consistent with prior research indicating that SBT provides students the opportunity to actively engage in practice by addressing scenarios similar to real-life situations, thus allowing them to hone their nursing, communication, critical thinking, self-efficacy and clinical decision-making skills [24–27]. Additionally, a meta-analysis of 15 studies supports the conclusion that SBT can effectively facilitate the transition from a learning environment to clinical practice because it can help students’ to enhance their knowledge, professional skills and clinical practice competencies [28]. Given that SBT can effectively prepare students for clinical practice, it should be offered before clinical practice.

This study identified three advantages of simulation compared with clinical placement. First, the students valued having tangible opportunities to take on clinical nursing leadership roles, as clinical placements often restrict them to an observer role to ensure patient safety [29]. Second, the students found the debriefing sessions to be particularly helpful because they were given the opportunity to ask questions, reflect on their experiences and identify areas for improvement [29, 30]. Third, the students appreciated the opportunity to learn by trial and error without harming patients. Through learning from their mistakes, students can learn flexibly and develop durable skills; the experience also can be quickly recalled if they face the same scenario in a real-life setting in the future. In this way, students can transfer learning from the simulation to their future clinical practice [31].

The majority of students considered SBT to be a viable alternative in situations where clinical practice is not feasible, such as during the COVID-19 pandemic. Otherwise, they argued that SBT could not fully replace clinical placement due to several limitations, including differences in the facilities and procedures and the challenges of accurately replicating certain aspects of clinical training. The students provided suggestions for improving the design of SBT to better mirror a real clinical environment. For example, some students suggested involving clinical nurses as facilitators, a practice supported by other research [32, 33]. To ensure high-quality simulation standards, simulationists are recommended to not only undergo accreditation and certification training but also participate in continuing education programmes to remain current with the latest clinical protocols [34].

The facilities and equipment available in simulation training laboratories also played a vital role in the students’ simulation experience. In this study, students noted discrepancies between the resources available in the laboratory and those in real clinical settings. This limitation was also highlighted in previous research [35]. However, it is very costly to set up and maintain a simulation laboratory. Each nursing institution should develop a financial plan with an annual budget allocation for simulation laboratories, and should actively apply for funding to support the facilities and equipment [35].

The students in our study emphasised the importance of allowing ample time for briefing so that they could fully understand, discuss and prepare to perform the simulation scenario. If expanding the briefing session is not feasible, a pre-briefing could be arranged up to four weeks prior to participation in the scenario, using various methods such as lectures and visits to high-fidelity laboratories [36]. The duration of pre-briefing may range from 5 to 30 min depending on the learning objectives and the students’ needs and level of experience [37]. Simulationists should provide information about as the content of the scenario and their expectations regarding the learners’ performance during the pre-briefing session [38]. Finally, our findings suggest that the variety and complexities of case scenarios used in SBT should be expanded to better replicate real clinical situations. Additional simulation sessions may be required if SBT is intended to replace clinical placements.

### Limitation

The study sample was drawn from only one government-funded public university in Hong Kong. Contextual factors such as faculty expertise, curriculum integration of simulations, access to high-quality simulation facilities, students’ background and focusing on a single specialty area may have influenced the study results; therefore, the applicability of the findings to other universities in Hong Kong and other regions or countries is uncertain. However, the findings of this study provide some important insights regarding nursing education. Further studies should explore the use of SBT as a substitute for clinical placement in other speciality areas and broader variety of institutions using a longitudinal design. On the other hand, the use of convenience sampling may be subject to self-selection bias as more motivated students are more likely to participate, thereby limiting the generalisability of the study findings to the entire study sample. Additionally, we recruited the instructors to moderate the focus group interviews with their respective simulation groups due to resource constraints. This might have introduced social desirability bias: the students may have tended to provide favourable responses to their instructors and to avoid expressing negative perceptions or experiences. To address this limitation, we assured the students that their participation in the interview would not affect the assessment of their participation in the simulation.

## Conclusion

This is one of the first qualitative studies in Hong Kong to explore the perceptions of pre-licensure nursing students regarding the use of SBT as a substitute for clinical practice. Our study findings indicate that students perceive SBT as complementary rather than as a full replacement for clinical placement. To refine the SBT, students suggested extending the briefing, improving case scenario complexity and patient diversity to better replicate real clinical situations, and upgrading laboratory facilities to include more realistic equipment. Future studies should explore the potential of SBT as a substitute for clinical training in other speciality areas and broader variety of institutions using a longitudinal design.

## Supplementary Information

Below is the link to the electronic supplementary material.

Supplementary Materials 1 and 2

## Data Availability

Data are available upon reasonable request from the corresponding author.

## Abbreviations

A&E: Accident and Emergency
BN: Bachelor of Nursing
MNSP: Master of Nursing Science (Pre-registration)
NLN: National League for Nursing
SBT: Simulation-based training
SD: Standard Deviation
TUNS: Temporary undergraduate nursing student in the public hospitals
US: United States
