# Supplementary material for: Perception of simulation-based training as a replacement for clinical practice training among pre-licensure nursing students: a qualitative study
Source: BMC Nurs. 2025 Dec 1;24:1456. doi: 10.1186/s12912-025-04096-4 (PMC12667138; doi:10.1186/s12912-025-04096-4)
Supplement: Supplementary file 1 — Supplementary Materials 1 and 2 [file 12912_2025_4096_MOESM1_ESM.docx]

**Supplementary Material 1** Focus group interview guide

**Preparation**

1. Introduce the interviewer.
2. Explain the study and obtain their consent.
3. Assign a specific number to each participant.

- The number will be used to call them during the interview to ensure anonymity.
- Every time before they speak up, please report their number for easy identification in the audio-record.

**Semi-structured interview**

1. What is your perception of simulation-based training (SBT)?
2. What’s your views on replacing some clinical training hours by SBT?
3. What is the most pleasurable aspect of your experience with SBT?
4. What is the most difficult or unpleasant aspect of your experience with SBT?
5. To what extent SBT facilitates the development of your clinical competency?
6. What are the areas that are likely to be the most challenging to your competency in providing patient care?

Follow-up prompts will be used to elicit in-depth information. For example:

- Can you tell me more about…?
- How did that happen…?
- How did you feel about that?

**Round up**

At the end of the interview, the interviewer will ask participants if they have any additional information. Then, the interviewer will round up the interview contents.

**Supplementary Material 2** Themes, subthemes, codes and sample quotes

| Theme | Subtheme | Code | Sample quote |
| --- | --- | --- | --- |
| Effective preparation for clinical practice | Bridging the gap between theoretical knowledge and practice | Realistic case scenarios (=18) | BN-G4S2: I think one of the great things about the A&E simulation is that it’s structured, so you always know what’s going on. I remember one simulation about a drug overdose where we had to give charcoal... Then when I was in A&E, I observed a real case of drug overdose which the patient needed to drink charcoal. That was really good simulation experience for handling drug overdoses. It helped me understand what the nurses were doing in that situation. In A&E, the nurses usually don’t explain what they are doing. We learned through observation. Since we covered that simulation case, I felt like I got a better view of what was happening.  MNSP-G2S4: When I had my clinical practicum at [hospital name] hospital, the resuscitation room was relatively small. The cases that I encountered weren’t very traumatic. However, I practised handling a trauma case in the simulation lab. At least we got to experience different scenarios that are common in A&E. It gave us a clearer understanding of the protocols or what nurses need to do. It’s a good experience. |
|  |  | Active participation (n=28) | MNSP-G1S2: I wouldn't call it the happiest experience, but it is definitely the most memorable. As Student 4 mentioned earlier, A&E is different from the wards, where routines are more common. In A&E, we often only get to observe, but there’s much more hands-on involvement in simulation. You have to go through the steps one by one, and I think I remember them better after doing it myself. If you just observe, you forget quickly. This kind of learning can only happen during simulations, which makes it a very memorable experience.  BN-G4S5: I think one of the good things about simulation is that we can take on the IC [in-charge] role to handle a case. In clinical practicum, we don’t really get that chance. But once we graduate, we’ll be an IC and need to manage everything. Therefore, the simulation allows us to practice that role without doing harm to the patient. I think this is helpful because it lets us experience the stress and panic feeling that come with being an IC nurse. |
|  |  | Opportunities to trial and error (n=30) | MNSP-G1S5: I think the most interesting aspect of the simulation lab is that it allows you to trial and error. In real life, we would not have the opportunity to do so, but in this simulation lab environment, we can afford making mistakes with minimal consequences. From that, you can learn what you should do next time and what you should not do.  BN-G1S5: It is okay to make mistakes because after the simulation, the tutor gives us a debriefing, telling us what we did wrong and how we can do better. When we reflect on our mistakes from the simulation, it helps us understand things better, so we won’t make the same mistakes when we’re in a real clinical setting later. |
|  |  | Reflective learning (n=30) | MNSP-G4S1: The most pleasurable aspect of this experience is the constructive feedback that I got from both my classmates and the tutor. They helped me understand how to improve and what areas to focus on. Their feedback is helpful for my future clinical practice. In real placements, not every clinical staff can give constructive feedback. Even if they give you feedback, their attitude can sometimes trigger hard feelings. I think tutors communicate with us more professionally so that it doesn’t end up with hard feelings. Instead, they help you realise, "Oh, I did well in some areas," which motivates you to improve. In reality, clinical staff often don’t have the time to give this kind of debriefing. |

| Theme | Subtheme | Code | Sample quote |
| --- | --- | --- | --- |
| Effective preparation for clinical practice | Improving skills | Case management (n=39) | MNSP-G2S2: I think simulation can enhance my clinical competency because it allows me to practice the entire patient journey, from admission to transfer to another hospital or discharge. We can get involved in different roles and procedures. During clinical placements, we only get to help with bits and pieces, like passing tools or hanging IV [intravenous] fluids. However, in simulation, we can get involved from the very beginning of the patient journey, like communicating with the ambulance staff via telephone. This would not happen in placements.  BN-G1S4: I think the main difference between the simulation and the real A&E setting is that it’s not about saving lives in simulation. Instead, it's about learning how to manage situations step by step, like what to do when someone has tachycardia. We learned what medications to administer and the appropriate timing for each step. The tutor takes us through each part, so we can really understand the guidelines and protocols. This way, I can learn more effectively without feeling overwhelmed. |
|  |  | Communication skills (n=13) | BN-G2S3: My communication skills have improved. As I mentioned earlier, besides communication with team members, we had to explain cases to the doctor. It made me realised how important it is to communicate effectively with the doctor about every order. This reminded us that in A&E or other wards, every order—big or small—must be communicated clearly, so that everyone understands, and we can minimise communication errors …We also learned to present this information systematically to avoid confusion. |
|  |  | Clinical decision-making skills (n=34) | MNSP-G3S2: Simulation lab allows us to apply what we’ve learned from books. I feel that in the lab, the teacher does most of the demonstrations while in the simulation lab, we take on a more active role. It really depends on the teacher's style... I’m not sure. But the simulation lab enhanced our decision-making skills compared to just read the textbook. We got to know the flow. If we are questioned [by clinical staff] about how to handle a case during clinical placements, like 'What should you do first?', we might feel less confused. |
|  |  | Nursing skills (n=10) | BN-G1S3: I think the simulation lab can help us develop proper nursing skills because the stressful environment in the ward doesn’t allow for comprehensive training. For example, when we do wound dressings, we might not have time to set up a sterile field. However, in the lab, we have more space and time to learn the correct procedures. In this way, we understand how things should ideally be done, rather than just trying to save time with the patient. With more standard practice in the lab, we won’t pick up improper skills during clinical placement.  MNSP-G2S4: I think the best part of the simulation lab, or my most enjoyable experience, is that I can apply the skills I've learned in lectures or practiced in labs. |
|  | SBT as an alternative to clinical practice in special circumstances | Special circumstances (n=18) | BN-G2S4: Under COVID, students were not allowed to observe many procedures, such as giving over 6L of O_2_ [oxygen], CPR [cardiopulmonary resuscitation], tubing, and others. I am thankful that at least I attended the simulation lab. Although I couldn't practice in real A&E settings, at least I learned how the procedures work. I understand what my role as a nurse would be, and I know what procedures the doctors typically perform and what we need to prepare. Even though I didn’t have the chance to see it in real life, the simulation lab provided a good experience. I know that if I become one of the nursing staff in the future, I won’t panic. |

| Theme | Subtheme | Code | Sample quote |
| --- | --- | --- | --- |
| Limitations of SBT in replacing clinical practice | Differences in procedures and facilities | Procedural differences（n=9） | BN-G4S5: In the simulation, it’s still somewhat theoretical since we're in a school setting. For example, the Valsalva maneuver—blowing air. In A&E, nurses typically avoid it in similar cases because it's often ineffective. Nine and a half out of ten times, they find it useless, so they don’t waste time on it. They just go straight to cardioversion. That is different.  MNSP-G1S5: I feel that what we learn in the ward differs from our training in the simulation lab. In the ward, the staff or mentors may teach us more effective methods to achieve the same results. This prepares us better for our career, as you'll learn to use more efficient methods for procedures. However, the simulation lab focuses on proper and traditional methods, which might be a bit more time-consuming. This means we learn two different perspectives: we understand how things should theoretically be done, while also discover quicker ways to achieve the same principles in practice. |
|  |  | Differences in facilities and equipment (n=25) | BN-G3S4: The staff organises workshops for fresh graduates, and they welcome students to join. From that, I heard them explain how to assess injuries to the hand or spine, or how to deal with dislocations. They had many different tools for temporary fixation and reduction for us to practice with. After that, they taught us how to use them in critical situations to prevent further injuries. They went over everything with us again and demonstrated how to use them. These hands-on experiences can only be found in a hospital setting. |
|  | Challenges of replicating certain aspects of clinical training | Clinical atmosphere (n=34) | BN-G4S5: The difference between the simulation and the actual A&E environment is mainly the atmosphere. It feels very different. In the simulation, we all know it’s not real, so the tension is missing. However, in real situations, everything moves much faster. I remember that during our simulation, everyone was more relaxed because we knew we weren’t dealing with a real patient, so there wasn’t as much urgency. Plus, since we were unfamiliar with the situation, we hesitated more, which slowed us down. Also, the environment itself isn’t the same; for instance, the oxygen cylinder and the cardiac monitor are just props, which makes it harder to fully immerse ourselves in the experience. |
|  |  | Interaction with real patients and families (n=36) | MNSP-G2S2: What I don’t like is that we’re working with a fake person. It feels like... (I: Is it because there’s no sense of realism?) Yes, exactly. I think that since it’s just a fake patient, even if I can’t do something, it doesn’t seem like there would be serious consequences. Therefore, I feel that my sense of responsibility isn’t as strong as it would be with a real patient.  MNSP-G1S4: I think simulation can never fully replace clinical practicum because real situations are often more complex. In the simulation lab, there are specific learning objectives, and teachers often add scenarios to make sure we understand key concepts. However, in real clinical settings, things don’t always go as planned, and we can’t predict what a person has been through. While simulations can give us a general idea of how procedures work, they can’t prepare us for real interactions with patients, like comforting them or dealing with family members who have questions. Those are experiences that can’t be truly replicated in a simulation. |
|  |  | Patient flow (n=33) | BN-G1S3: In the simulation lab, the focus is mainly on nursing skills. However, in real clinical setting, there are many paperwork tasks to handle. For example, when admitting a patient, we might need to call the family for information or fill out various system forms that we may not be very familiar with. In the simulation lab, we might not experience this, so we’ll have to learn how these systems work as we start working as a registered nurse.  BN-G2S1: The job of an A&E nurse involves so many tasks that can’t be fully demonstrated. For example, the entire flow from triage… after triage, waiting for the doctor to assess the patient, and borrowing equipment. That involves a lot of communication and preparation. It’s not just about the patient; it also involves the environment and equipment. Many of these subtle details can’t be replicated in a simulation. |

| Theme | Subtheme | Code | Sample quote |
| --- | --- | --- | --- |
| Limitations of SBT in replacing clinical practice | Challenges of replicating certain aspects of clinical training | Patient variety and complexity (n=27) | BN-G4S5: The downside of simulations is that in the simulation cases, as mentioned earlier, we already know the diagnosis. However, in real clinical setting, we don’t have that information. Additionally, a patient may present with multiple issues at the same time. For example, if a patient comes in from a road traffic accident, they could have multiple problems, including both trauma and medical issues. This complexity can’t be replicated in simulations.  MNSP-G4S1: We face the challenge of integrating our knowledge at school. A case might involve both cardiovascular and respiratory issues, but how do we identify it? In school, we have a clear topic for this case, so we understand it well. However, in real situations, things can be very complicated (Interviewer: multiple comorbidities). Exactly. If we focus only on one system, we won’t have enough hands-on experience when we go out. When we encounter multiple disciplines in real life, we might not know which tasks to prioritise. I think the case scenarios need to be more realistic. Perhaps we should start with foundational learning and then learn how to integrate everything later, making it closer to real-life situations. |
|  |  | Unpredictability (n=16) | BN-G3S3: I had a particularly unique experience in a real A&E setting at [hospital name] Hospital. After I attended a simulation session, I went to that A&E. There I witnessed two resuscitation rooms were operating simultaneously, but the computer system, similar to IPMOE [Inpatient Medication Order Entry], was down. It took about five minutes just to load a page. These situations are quite urgent and difficult to predict in real life. If we only relied on simulations, we wouldn't have these kinds of unique experiences.  MNSP-G2S3: I believe simulations provide a good foundation for us to familiarise ourselves with the operations of different specialties. However, in simulations, many factors are under control, like patients’ conditions and the absence of family members. The situations are all within a controlled environment. But after going through some placements, we know that it can be quite chaotic in real clinical settings. I think if we only rely on simulations, we won’t be able to handle real-life situations. Additionally, we haven't seen how other staff such as nurses and doctors manage these scenarios. If we suddenly get into the real clinical settings, we have no idea how to handle it. |
| Refining SBT | Design of SBT | Enhance the briefing (n=15) | BN-G2S4: I think the cases could be paced a bit slower or give us more time to understand each case. Maybe we could have five to 10 minutes for grouping first, since everyone may not be very familiar with what they’re doing. It would be helpful if there were some briefings to allow us to practice, or give us a little time to discuss with the tutor what we should know. If we know too little, everything can become chaotic, which ruins the entire learning experience. |
|  |  | Involve clinical nurses (n=7) | BN-G4S5: Both teachers are not very familiar with A&E setting, including some of the equipment. Their way of teaching is different from real clinical practice. A big issue with the simulation is that if the facilitators don’t know the practice well, they might give us incorrect information. However, the clinical nurses in A&E do these tasks every day. Even if their methods are slightly different from standard practice, they haven’t had any serious incidents so far. The difference lies within safe practices. |
|  |  | Increase case variety and complexity (n=25) | BN-G1S4: Different diseases and situations are important because, even after we graduated, we might not work in that specialty. Although there will be training later on, I think having different experience would be beneficial. This time, we only had two days of simulation. If we could have a few more days covering different diseases and situations, it would be much better. If simulation is used to replace clinical practice, there should be several weeks of exposure to different diseases and conditions. I believe we could learn a lot more from that. |
|  | Upgrading facilities and equipment | Upgrading facilities and equipment (n=6) | BN-G1S2: I think the simulation lab could be more like a ward setting, such as using systems like IPMOE [Inpatient Medication Order Entry], or CMS [Clinical Management System]. When we are in the ward, we need to interact with the staff who are using these systems. If the simulation lab could have a similar system or even a screen capture that shows us the buttons and their functions e.g. medication distribution or X-ray records etc. It would feel much more realistic, like we're actually in a ward. |

A&E: Accident and Emergency; BN: Bachelor of Nursing; G: Group; MNSP: Master of Nursing Science (Pre-registration); S: Student; SBT: Simulation-based training
